# Opioids Contribute to Fracture Risk: A Meta-Analysis of 8 Cohort Studies

**DOI:** 10.1371/journal.pone.0128232

**Published:** 2015-06-01

**Authors:** Zhaowei Teng, Yun Zhu, Feihu Wu, Yanhong Zhu, Xiguang Zhang, Chuanlin Zhang, Shuangneng Wang, Lei Zhang

**Affiliations:** 1 Department of Orthopedic Surgery, The People’s Hospital of Yuxi City, The 6th Affiliated Hospital of Kunming Medical University, Yuxi, Yunan, China; 2 Department of Nephrology, The People’s Hospital of Yuxi City, The 6th Affiliated Hospital of Kunming Medical University, Yuxi, Yunan, China; 3 Department of Anesthesiology, The People’s Hospital of Yuxi City, The 6th Affiliated Hospital of Kunming Medical University, Yuxi, Yunan, China; 4 Trauma Center of Yunnan Province, The Second People's Hospital of Yunnan Province, Kunming, Yunan, China; Van Andel Institute, UNITED STATES

## Abstract

**Objective:**

To evaluate the association between chronic opioid use for non-cancer pain and fracture risk by conducting a meta-analysis of cohort studies.

**Methods:**

Cohort studies were identified by searching PubMed and EMBASE from their inception to July 2014. A fracture was considered an endpoint. The information was extracted by two authors independently. When the heterogeneity was significant, a random-effects model was used to calculate the overall pooled risk estimates.

**Results:**

Eight cohort studies were included in the final meta-analysis. On the basis of the Newcastle-Ottawa Scale (NOS), six studies were considered to be of high quality. The overall combined relative risk for the use of opioids and fractures was 1.88 (95% confidence interval [CI] 1.51-2.34). A subgroup analysis revealed the sources of heterogeneity. The sensitivity analysis indicated stable results, and no publication bias was observed.

**Conclusions:**

This meta-analysis of cohort studies demonstrates that opioids significantly increase the risk of fractures.

## Introduction

The World Health Organization estimates that at least 20% of individuals worldwide have varying degrees of chronic pain [1]. Opioids, which provide effective pain relief in a range of persistent non-cancer pain conditions, are widely and increasingly used for their analgesic and psychotropic effects [2]. An epidemiological study of chronic, non-malignant pain in Denmark revealed that nearly 3% of the Danish population used opioids regularly [3]. Chronic exposure to opioids is frequently encountered in clinical practice. Chen’s study [4] showed a lack of correlation between changes in opioid dose and clinical pain scores in a group of chronic pain patients regardless of the clinical pain conditions for which opioid therapy was intended. However, a large increase in opioid use has occurred in the USA, with more than 3% of persons 70 years and older in the U.S. estimated to be regular users of opioids [5]. In addition, the misuse of opioids is the fastest growing form of drug misuse and is the leading cause of accidental overdose and mortality [6]. Because pain is the fifth vital sign in the USA, there has been increasing attention paid to the use and effects of opioids. We know that approximately 80% of patients taking opioid therapy will experience an adverse effect, such as constipation, hypogonadism or the suppression of the innate and acquired immune systems; thus, considerable controversy remains regarding the use of opioids to treat persistent non-cancer pain [2]. Some previous studies have reported an association between opioids and fracture risk [2,7–9], although these studies have failed to demonstrate a significant increase in fracture risk when using opioids. However, a trend toward a higher fracture risk with the use of opioids was found [4,10]. A previous meta-analysis [11] demonstrated that a relative fracture risk was associated with several classes of psychotropic drugs, including opioids. However, only six studies on opioids were included in this analysis, which did not allow firm conclusions to be drawn because of the potential of heterogeneity and publication bias.

Opioids are widely used for non-cancer pain, and to our knowledge, no specific meta-analysis of the association between fracture risk and opioid use has been conducted to date. Therefore, we performed a meta-analysis with the purpose of assessing the fracture risk among opioid users. In this study, we followed the Meta-analysis of Observational Studies in Epidemiology (MOOSE) guidelines [12].

## Materials and Methods

### Search strategy and data sources

We searched MEDLINE (PubMed) and EMBASE (1947 to 2014 July 21) for cohort studies describing the association between opioid use and fracture risk without restrictions. We also searched the bibliographies of relevant articles to identify any additional studies. We used the following search terms: (i) fracture*[Title/Abstract] OR “Fractures, Bone”[Mesh]; (ii) opioid*[Title/Abstract] OR “Analgesics, Opioid”[Mesh]; and (iii) cohort study OR "Cohort Studies"[Mesh].

### Study selection

Studies were considered eligible if they met all of the following criteria: (i) presented original data from a cohort study; (ii) evaluated the association of opioid use with fracture incidence; (iii) had opioids as the exposure of interest; and (iv) provided hazard ratios (HRs) or the adjusted relative risks (RRs) and the corresponding 95% confidence intervals (CIs). If the data were duplicated or the population was studied in more than one study, we included the study with the largest sample size and the most comprehensive outcome evaluation.

### Data extraction and quality assessment

Two investigators (ZWT, YZ) independently evaluated the eligibility of the studies retrieved from the databases based on the pre-determined selection criteria. In addition, a cross-reference search of eligible articles was conducted to identify studies not found in the computerized search. These two authors independently extracted the following data: the first author’s name; year of publication, patients’ ages, cohort size, study regions, years of follow-up, study design, HR or RR and the 95% CIs, and statistical adjustments for confounding factors. Any disagreements were resolved either by discussion or in consultation with the co-corresponding author (XGZ). The methodological quality assessment was based on the Newcastle-Ottawa Scale (NOS) [13]. The maximum NOS score was 9. We defined low quality as a Newcastle-Ottawa Scale score < 7.0 and high quality as a score ≥ 7.0.

### Statistical analyses

We investigated the association between the use of opioids and the risk of fracture by using adjusted data for the main analyses. We computed a pooled RR and 95% CI from the adjusted RRs or HRs and 95% CIs reported in the studies. The HRs were considered to correspond to RRs. The Cochran Q and I^2^ statistics were used to evaluate the statistical heterogeneity [14]. When the P value was < 0.1 and the I^2^ value was > 50%, the data were considered to be heterogeneous, and a random-effects model (DerSimonian and Laird method) [15] was applied because it represents a more conservative approach to the calculation of a weighted estimate effect using an RR. Otherwise, a fixed-effects model [16] was used to estimate the overall summary effect sizes when no heterogeneity was present in the included studies. To further explore the origin of heterogeneity, we also performed subgroup analyses by study design, study region and fracture type (any fracture, with all fracture types combined, and hip fractures).

To assess the stability of our results, a sensitivity analysis (by excluding each single study in turn) was conducted to estimate the influence of individual studies on the pooled result.

We used Egger’s test (linear regression method) [17] and Begg’s test (rank correlation method) [18] to assess the potential publication bias.

## Results

### Literature search and study characteristics

**Fig 1:**
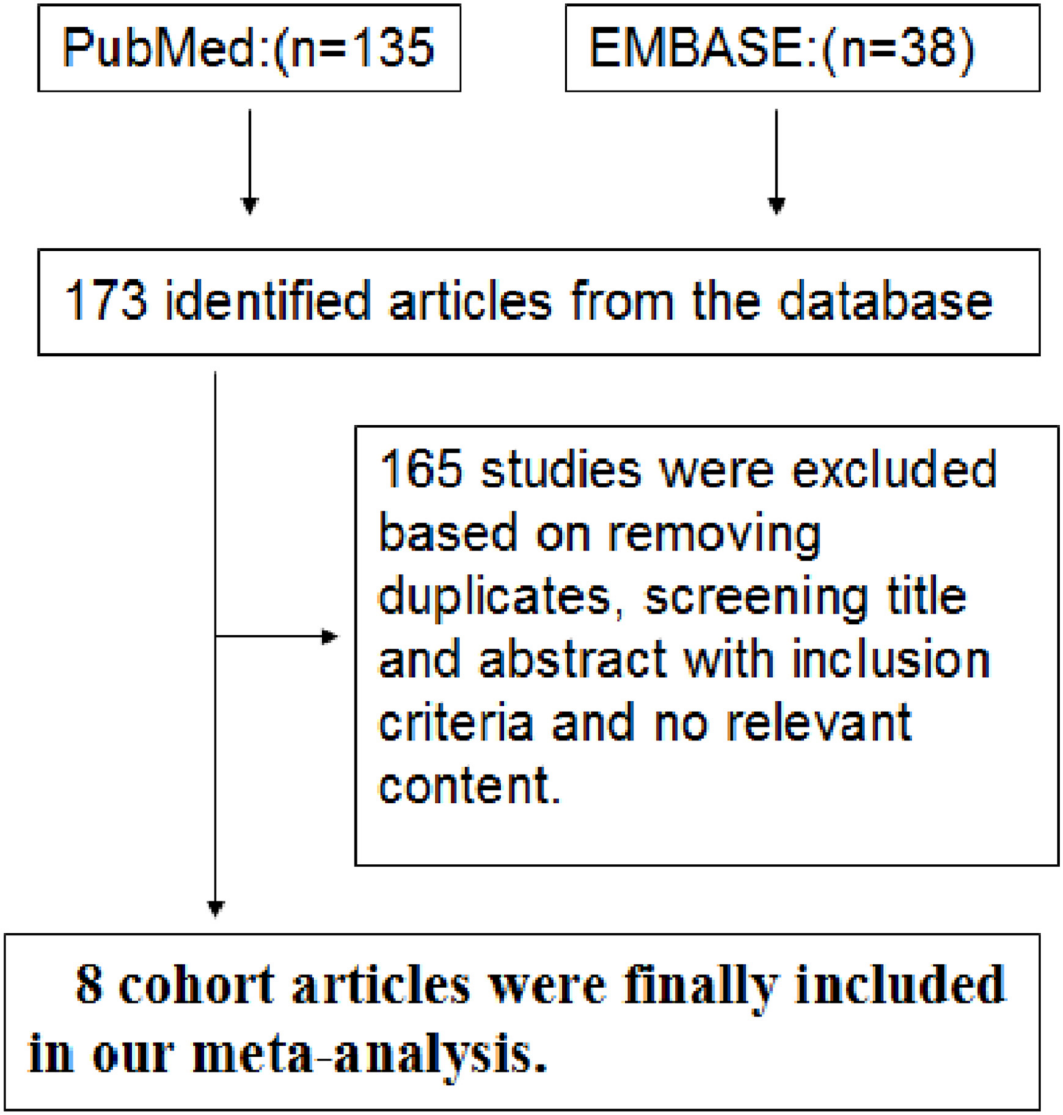
Flow Chart Illustrating the Literature Search for Cohort Studies on Opioids in Relation to Fractures.

A total of 173 articles were identified in the initial search. Of these articles, 165 were excluded after reviewing the titles and abstracts, removing duplicates, and thoroughly reading the full text. As a result, we included eight cohort studies in our final analysis (Fig 1) [10,19–25]. Five [10,21,23–25] of the 8 cohort studies were from the United States, and 3 studies were from other countries, namely, Sweden, England, and Denmark. The general characteristics of the eight studies and the quality scores for the studies are summarized in Table 1. Of the 8 studies, six were prospective studies and two were retrospective studies. Six were high-quality studies (scores ≥ 7.0) (Table 1).

**Table 1:** Characteristics of the 8 Cohort Studies included in the Final Analysis of Opioids and Fracture Risk.

Author, year; location	Age of the participants, years	Fracture type/assessment	Study design	Cohort size	Follow-up time	Models	Adjustment for covariates	NOS
Guo, 1998, Swedish	≥75	Hip/ICD-9	Prospective cohort	1608	4.4 years	Cox proportional hazards models	Age, gender, education, residence, ADL limitation, cognitive impairment, history of stroke and tumors	8
Kristine, 2003; USA	≥65	Fractures/radiology reports	Prospective cohort	8127	4.8 years	Cox proportional hazards models	Age, gender, race, health status, smoking, walking exercise, functional impairment, cognitive function, depression, weight change	9
Card et al., 2004; UK	NA	Hip/NA	Prospective cohort	99467	7.3 per 10000 person-years	Cox regression models	Age, gender, corticosteroid use	6
Sachin, 2006; USA	≥65	Hip/ICD-9	Prospective cohort	362503	464 days	Cox regression models	Age, gender, antidepressant use, antipsychotic use, anxiolytic /hypnotic use	7
Kathleen, 2010; USA	≥60	Fractures/ICD-9	Prospective cohort	2341	32.7 months	Cox proportional hazards models	Age, gender, smoking, depression, substance abuse, dementia, comorbidities, prior fracture, pain site, antidepressant use, sedative use, HRT /bisphosphonate use	9
Miller, 2011; USA	≥65	Fractures/ICD-9	Retrospective cohort	17310	451 per 1000 person years	Cox proportional hazards models	Age, gender, diabetes stroke, osteoarthritis, comorbidity index, stroke, diabetes	6
Vestergaard 2012; Denmark	45–58	Fractures/X-ray	Prospective cohort	2016	10 years	Cox proportional hazards models	Age, HT, BMI, baseline spine BMD, family or prior fracture, serum 25-hydroxy-vitamin D and smoking	9
Laura, 2013; USA	58.73±13.43	Lower extremity/ICD-9	Retrospective cohort	7447	3–8 years	Cox proportional hazards models	Age, race, completeness of SCI level and duration of SCI	7

Note: fractures indicate overall fractures; hip indicates hip fracture; NOS: Newcastle-Ottawa Scale

### Main analysis

**Fig 2:**
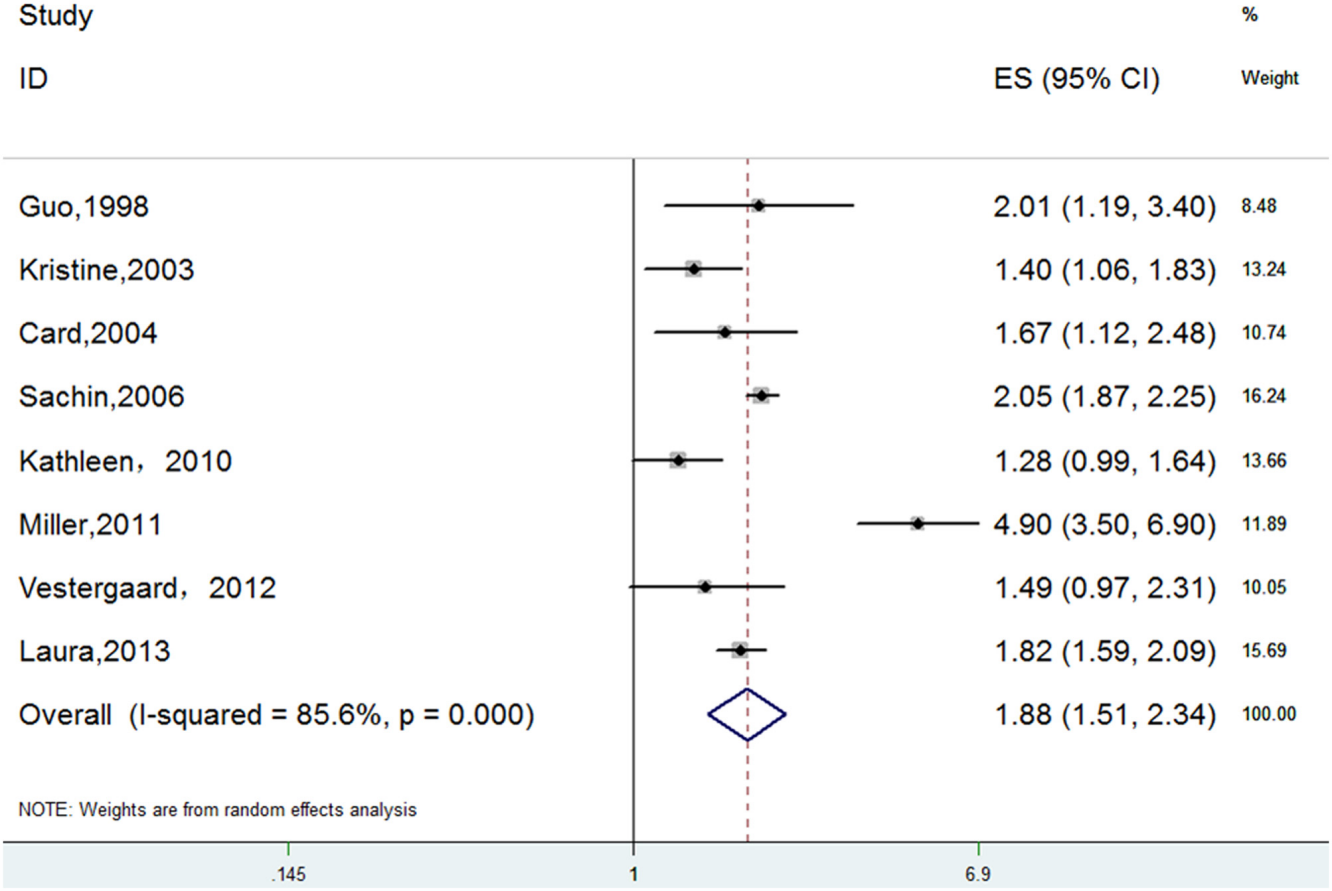
Forest Plot of RR with CI for Opioid Use and Fracture Risk.

The meta-analysis of the 8 cohort studies, which included 500819 individuals, indicated significant, positive associations between opioid use and overall fracture risk (RR 1.88, 95% CI 1.51–2.34). Substantial heterogeneity was observed (P = 0.000, I^2^ = 85.6%) (Fig 2).

**Fig 3:**
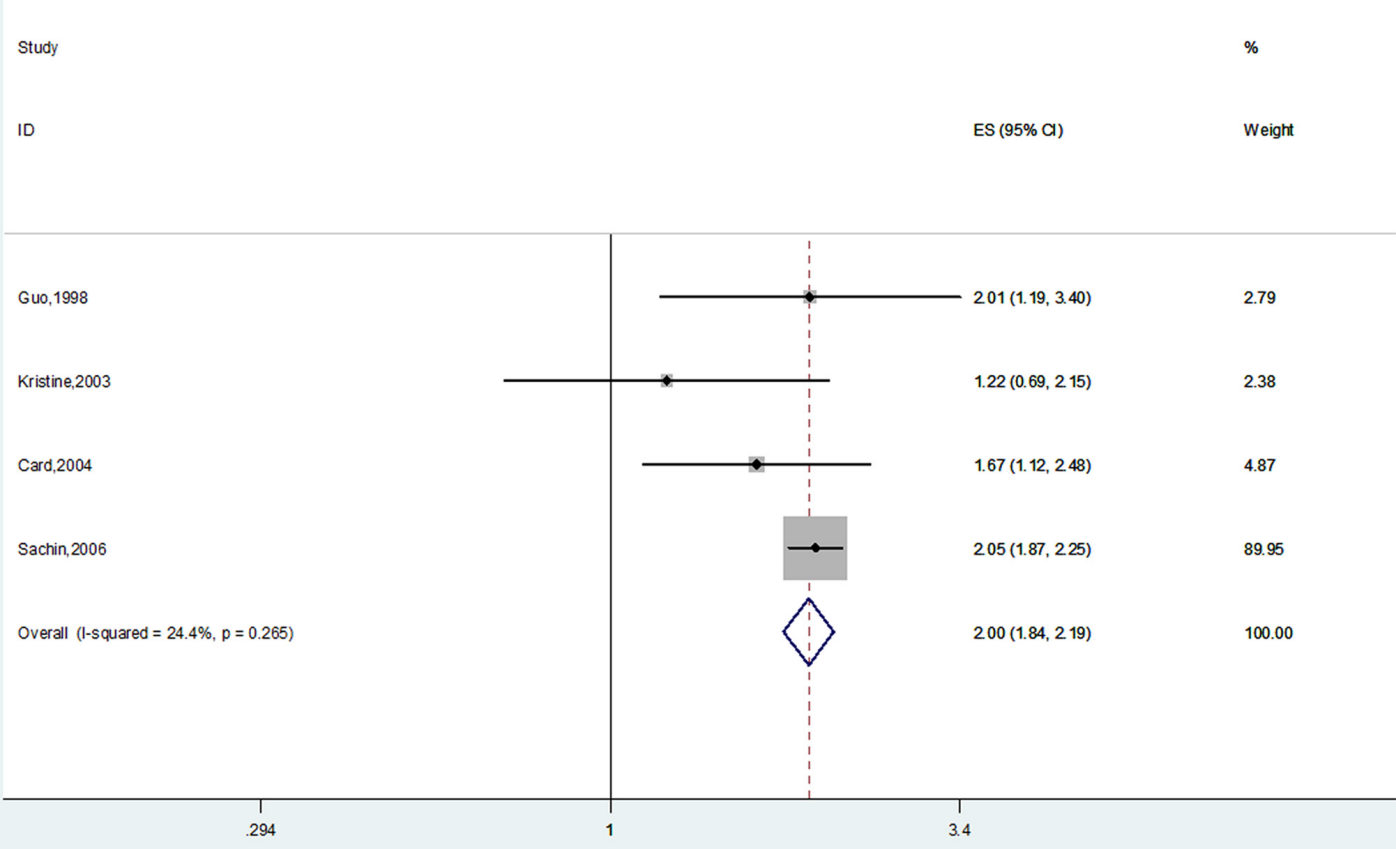
Forest Plot of RR with CI for Opioid Use and Hip Fracture Risk.

Data on opioid use and hip fracture risk were available for 4 studies [20–23]. The pooled results indicated that opioids contribute significantly to the risk of fracture (RR 2.00, 95% CI 1.84–2.19). No statistical heterogeneity was observed (P = 0.266, I^2^ = 24.4%) (Fig 3).

### Subgroup meta-analysis

A subgroup meta-analysis was performed according to the study design type. We observed a significant, positive association between opioid use and fracture risk in both prospective and retrospective cohort studies (Table 2).

**Table 2:** Subgroup Analyses of the Association between Opioid Use and Fracture Risk.

Factor		No. of studies	RR (95% CI)	Heterogeneity P (I^2^%)
Study design	Retrospective cohort	2	2.95 (1.12–7.78)	0.000 (96.4)
	Prospective cohort	6	1.62 (1.31–2.02)	0.003 (72.5)
Region	Europe	3	1.68 (1.30–2.17)	0.690 (0.0)
	USA	5	1.97 (1.49–2.61)	0.000 (91.4)
Fracture type	hip fracture	3	2.03 (1.86–2.22)	0.615 (0.0)
	any fracture	5	1.88 (1.51–2.34)	0.000 (91.0)

The subgroup analysis by region indicated a significant tendency toward increased fracture risk with the use of opioids. No statistical heterogeneity was observed in the European group (Table 2).

**Fig 4:**
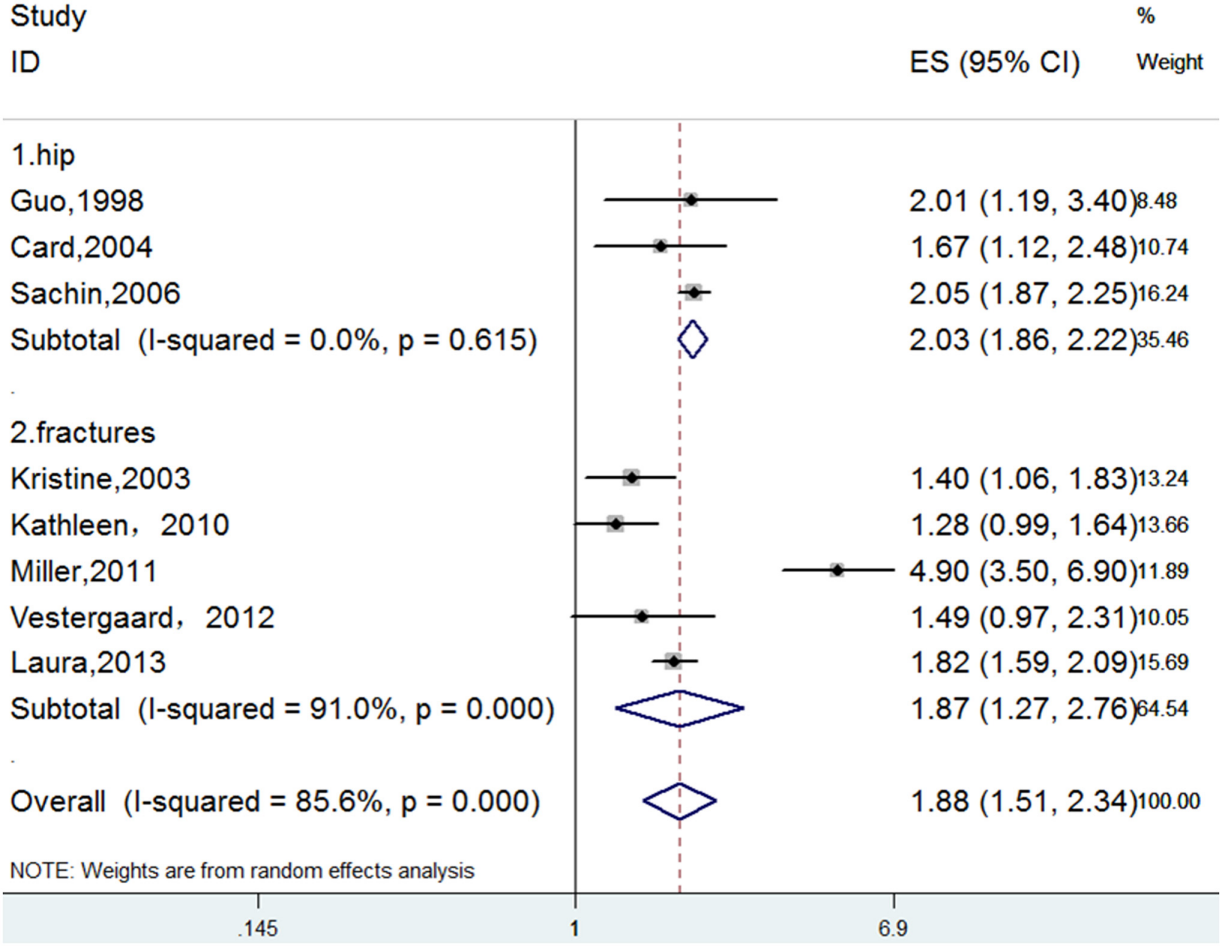
Forest Plot for a Subgroup Meta-analysis by Anatomical Fracture Site.

A subgroup analysis was performed according to the anatomical fracture site. We assigned studies that presented data on hip fracture to one group, and the other studies were assigned to another group. We also observed significant, positive associations between opioid use and the risk of hip fractures. No statistical heterogeneity was observed in the hip group (Fig 4, Table 2).

To identify the influence of the retrospective studies, we performed further analyses without two studies [21,25]. We found that when we eliminated the Miller [21] study, the pooled outcome was stable, and the heterogeneity was significantly decreased (RR = 1.52, 95% CI 1.25–1.85, I^2^ = 59.3%, P = 0.061). After we also eliminated the Laura [25] study, no heterogeneity was discovered (RR = 1.36, 95% CI 1.14–1.61, I^2^ = 0.0%, P = 0.805).

### Sensitivity analysis

To evaluate the robustness of our analysis, we conducted a sensitivity analysis by recalculating the pooled results of the primary analysis by excluding one study per iteration. The outcome revealed that the exclusion of any single study did not alter the overall combined result (Table 3).

**Table 3:** Sensitivity Analysis of the Association between Opioid Use and Fracture Risk.

Excluded study	RR (95% CI)	P (I^2^%)
Guo et al., 1998	1.87 (1.48–2.36)	0.000 (87.7)
Kristine, 2003	1.97 (1.56–2.49)	0.000 (86.1)
Card et al., 2004	1.91 (1.51–2.42)	0.000 (87.5)
Sachin, 2006	1.86 (1.37–2.52)	0.000 (86.6)
Kathleen, 2010	2.00 (1.60–2.50)	0.000 (84.2)
Miller et al., 2011	1.68 (1.44–1.96)	0.006 (67.2)
Vestergaard et al., 2012	1.93 (1.53–2.44)	0.000 (87.3)
Laura, 2013	1.90 (1.41–2.54)	0.000 (87.5)

Note: The values of P and I^2^ represent the heterogeneity.

### Publication bias

**Fig 5:**
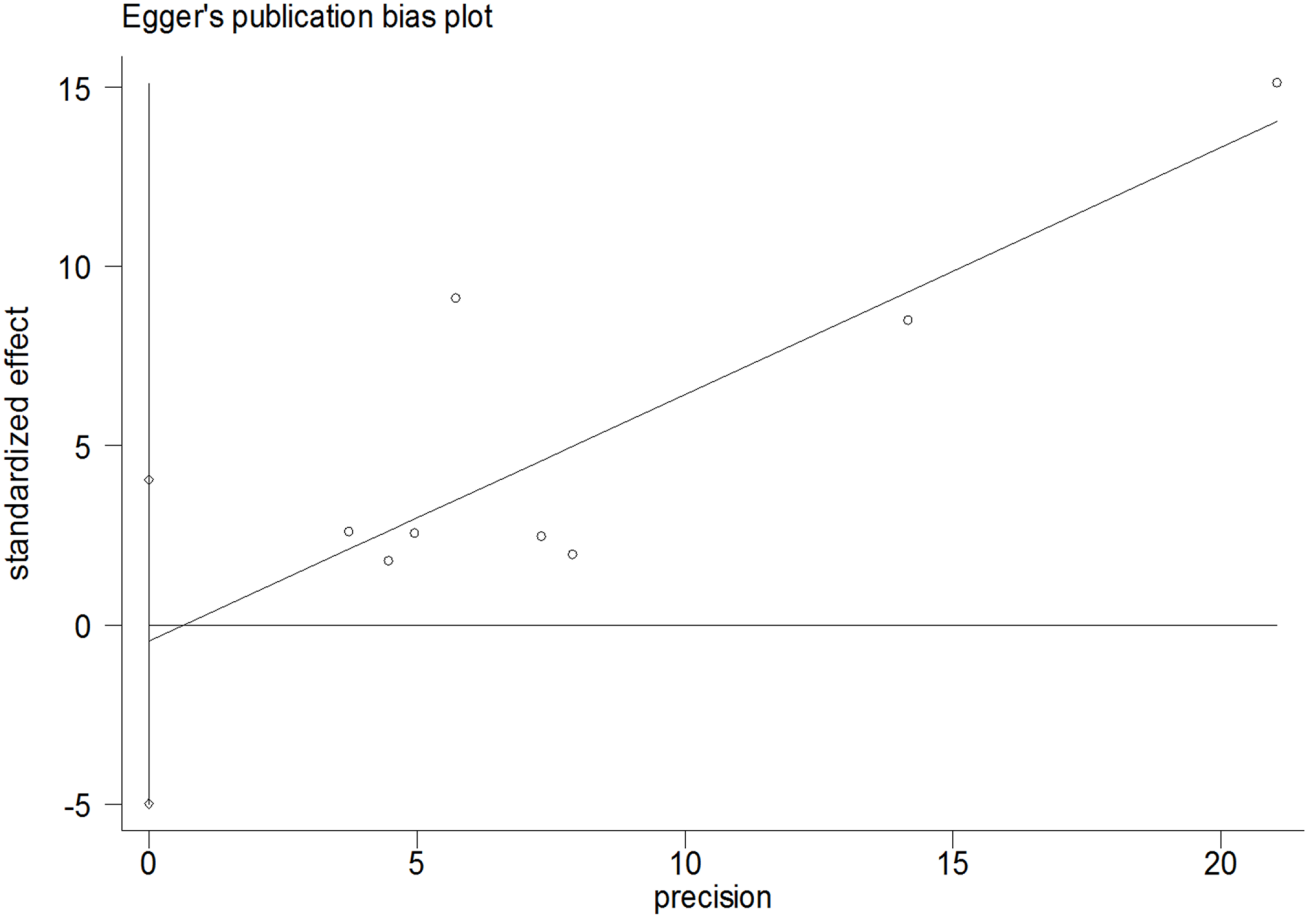
Egger’s Publication Bias Plot.

**Fig 6:**
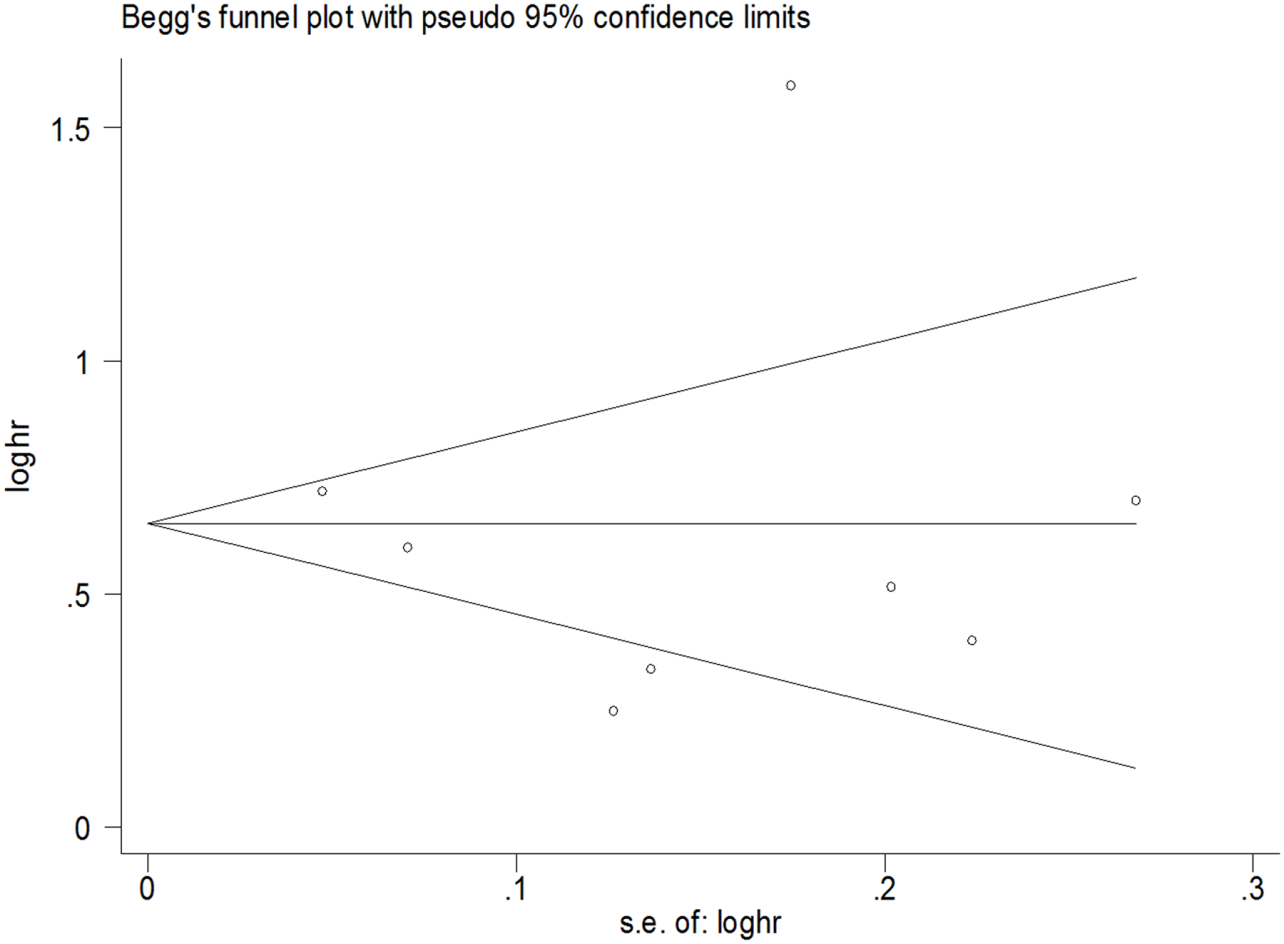
Begg’s Funnel Plot of the 8 Cohort Studies.

The Begg rank correlation test and Egger linear regression test indicated no evidence of publication bias among the studies [Begg, P > |z| = 0.902; Egger, P = 0.809, 95% CI -4.98–4.05] (Figs 5 and 6).

## Discussion

To the best of our knowledge, this is the first meta-analysis performed to describe the relationship between opioid use and fracture risk based on cohort studies. The primary findings from our study consistently suggest that opioids are associated with an 88% increase in fracture risk. When the fractures were restricted to the hip, the risk of fracture increased 2-fold. The findings are comparable to those of both a meta-analysis [11] and recent clinical trials [26,27] that demonstrated a significantly increased fracture risk associated with opioid use. In addition, our results are comparable to meta-analyses that analyzed other pharmaceutical agents and fracture risk. For instance, Eom’s study [28] showed that a significant positive association was observed between the use of selective serotonin reuptake inhibitors and the risk of fracture (OR 1.69, 95% CI 1.51–1.90), Bazelier et al. [29], using an individual patient data meta-analysis, reported a 1.2 to 1.5-fold increased risk of fractures for women using thiazolidinediones. Takkouche’s study [11] found that psychotropic medications may play an important modifiable role in the development of fractures. Studies on opioid medications have suggested that opioids are associated with a 38% increase in fracture risk. However, in Takkouche’s study [11], only six trials were included, and only three were cohort studies. Heterogeneity was present among the included studies, which may make the results unreliable. In the present study, although heterogeneity was also observed, the findings were stable and robust based on our sensitivity analysis. There are several plausible mechanisms by which opioids may increase fracture risk [30,31]. For example, adverse opioid effects, such as sedation and dizziness, can increase the propensity to fall due to central nervous system effects [32]. Opioids may decrease bone mineral density by impairing the production of endogenous sex steroids, and the effect on bone metabolism may directly weaken bone structure [32]. As elderly persons are at increased risk of developing osteoporosis and pain, the opioids used to treat pain in this population may increase the risk of subsequent fractures.

Although the relationship between opioid analgesics and fractures is well known [10,24,33,34], no convincing meta-analysis of this relationship has been conducted to date. For example, a previous meta-analysis [11] of the associations between opioid use and fracture risk did not obtain robust conclusions because of publication bias and study heterogeneity. However, in our meta-analysis, the combined result was stable and robust according to the sensitivity analysis, and publication bias was not observed.

Observational studies cannot prove causality [35]. However, a major strength of our study is that all of the included studies adopted a cohort design, and no other epidemiological observational studies were included. Six studies were prospective cohort studies that tracked patients for a long period of time. Most of the studies were of good quality and had large sample sizes and accurate outcome assessments. In addition, although a high I^2^ (85.6%) was present for overall fracture risk, we identified heterogeneity sources by performing subgroup analyses. In the subgroup analysis by study design type, the heterogeneity of prospective cohort studies was significantly reduced, which indicated that retrospective cohort studies may introduce heterogeneity. By performing a subgroup analysis based on study region, we found that studies conducted in the USA made major contributions to heterogeneity. Therefore, one possible reason for the heterogeneity may be the inclusion of studies conducted in different regions. In the subgroup analysis by overall fractures and hip fractures, we found that no heterogeneity was observed in the hip fracture studies, and all of the heterogeneity was derived from the overall fracture group. Two studies [21,25] played a key role in the heterogeneity. Therefore, study design and region increased the heterogeneity. Of course, many other factors may or may not have had additional contributions. We will evaluate these factors in the future when the necessary data are available.

Despite the advantages of this study, some limitations should be mentioned. First, although we searched all cohort studies describing the association of opioids with fractures, the eligible studies were restricted to the English language. The number of relevant studies was still relatively small, which implies that some studies may have been missed due to their publication in non-English language journals or publication in a book or a journal that is not included in the computer databases. Second, studies with nonsignificant results, especially those that show an absence of effect, may not be published because they are rejected by the journals or because the investigators are unwilling to submit them for publication [36]. Although we controlled for publication bias using statistical methods, publication bias could not be completely ruled out. Thus, the pooled effect measure may be overestimated. Third, the degree of control for confounding variables, such as age, gender, body mass index and comorbidity, also varied between studies. Fourth, in this meta-analysis, we were not able to investigate the effect of different opioid doses because relevant data were available in only a few studies. Fifth, it was unfortunate that we were not able to define contributing factors because all of the included papers were from Western countries and all of th pe participants were Caucasian. Therefore, our results cannot be generalized to worldwide populations, especially non-Western populations. As a result, more investigations of contributing factors, such as ethnicity, participant education level and socioeconomic class, especially in non-Western populations, are required. Sixth, we were not able to determine the fracture timing, whether medication sedation effects would be more likely shortly after starting opioid therapy, or whether metabolic biomechanical reasons would be more likely the longer the medication is taken. Thus, further research into fracture timing is needed. Finally, our meta-analysis is of good quality but is not the most comprehensive study because only cohort studies were included. Thus, higher quality and more comprehensive analyses are still needed as more data are published in the future.

## Conclusions

In summary, our meta-analysis of cohort studies demonstrates that opioids may play an important role in the development of fractures and that opioid use may significantly increase fracture risk. Further studies, including studies that are well-designed, international trials (especially prospective, non-Western studies), studies that examine the mechanisms responsible for the fractures due to opioid use and studies that aim to prevent these fractures from occurring are required to provide more convincing evidence for clinical practice and fracture prevention.
